# Os acromiale: prevalence and associated patient-related factors—a population-based study of three thousand and fifty participants

**DOI:** 10.1007/s00264-022-05406-0

**Published:** 2022-05-05

**Authors:** Cornelius Sebastian Fischer, Matthias Floß, Till Ittermann, Robin Bülow, Henry Völzke, Marc-Daniel Ahrend, Jörn Lange

**Affiliations:** 1 Department of Traumatology and Reconstructive Surgery, BG Unfallklinik Tübingen, Eberhard Karls University Tübingen, Schnarrenbergstraße 95, 72076 Tübingen, Germany; 2 Clinic of Trauma, Reconstructive Surgery and Rehabilitation Medicine, University Medicine Greifswald, Greifswald, Germany; 3 Institute for Community Medicine, University Medicine Greifswald, Greifswald, Germany; 4 Institute of Diagnostic Radiology and Neuroradiology, University Medicine Greifswald, Greifswald, Germany

**Keywords:** Os acromiale, Shoulder, MRI, Population-based, Associated factors

## Abstract

**Purpose:**

The presence of os acromiale is of clinical relevance before performing shoulder surgery but ethnic differences and little information regarding associated factors seem to be present. Population-based studies to clarify these topics are essential so the purpose of this study was to assess the prevalence, anatomy, and associations of os acromiale in a general adult population.

**Methods:**

Both shoulders of 3050 participants from the population-based Study of Health in Pomerania (SHIP) were assessed on magnetic resonance imaging (MRI). Associations with the os acromiale were calculated for sex, age, body height, body weight, and heavy mechanical oscillations on the upper extremity.

**Results:**

In total, 1.9% (58/3050) had an os acromiale, while 21 were unilateral left, 23 were unilateral right, and 14 were bilateral. Sixty-eight meso-acromions, three pre-acromions, and one meta-acromion were detected. Os acromiale were more frequent in men (right side: *p* = 0.037, left side: *p* = 0.005). Overall, no differences in sides (*p* = 0.808), to participants’ age (right: *p* = 0.993, left: *p* = 0.499), body height (right side: *p* = 0.241, left side: *p* = 0.154), and the exposure to heavy mechanical oscillations on the upper extremity (right: *p* = 0.054, left: *p* = 0.117) were detected.

**Conclusion:**

Our results support the genetic theory for the aetiology of the os acromiale due to the lower prevalence of the os acromiale in north-eastern Germany compared to the worldwide prevalence (1.9 to 7%) and the lacking association to lifestyle, age, gender, or sides. Additionally, it is important to be aware of possible os acromiale before surgery.

## Introduction

The os acromiale is an accessory bone of the acromion, resulting from a failure of fusion during the development of the acromial process. The acromion is already formed in the early development. Fealy et al. documented its consistent cartilaginous shape throughout all gestational periods starting from gestational week 13 [1]. During physiological growth, three independent centres of ossification, the pre-, meso-, and meta-acromion, are formed until the age of 18 who will grow and fuse with the basi-acromion to the definite acromion between the age of 23–25 years [2–4]. Recent studies on adolescents described a considerably earlier complete osseous fusion between the age of 14 and 16 [5, 6].

**Fig. 1:**
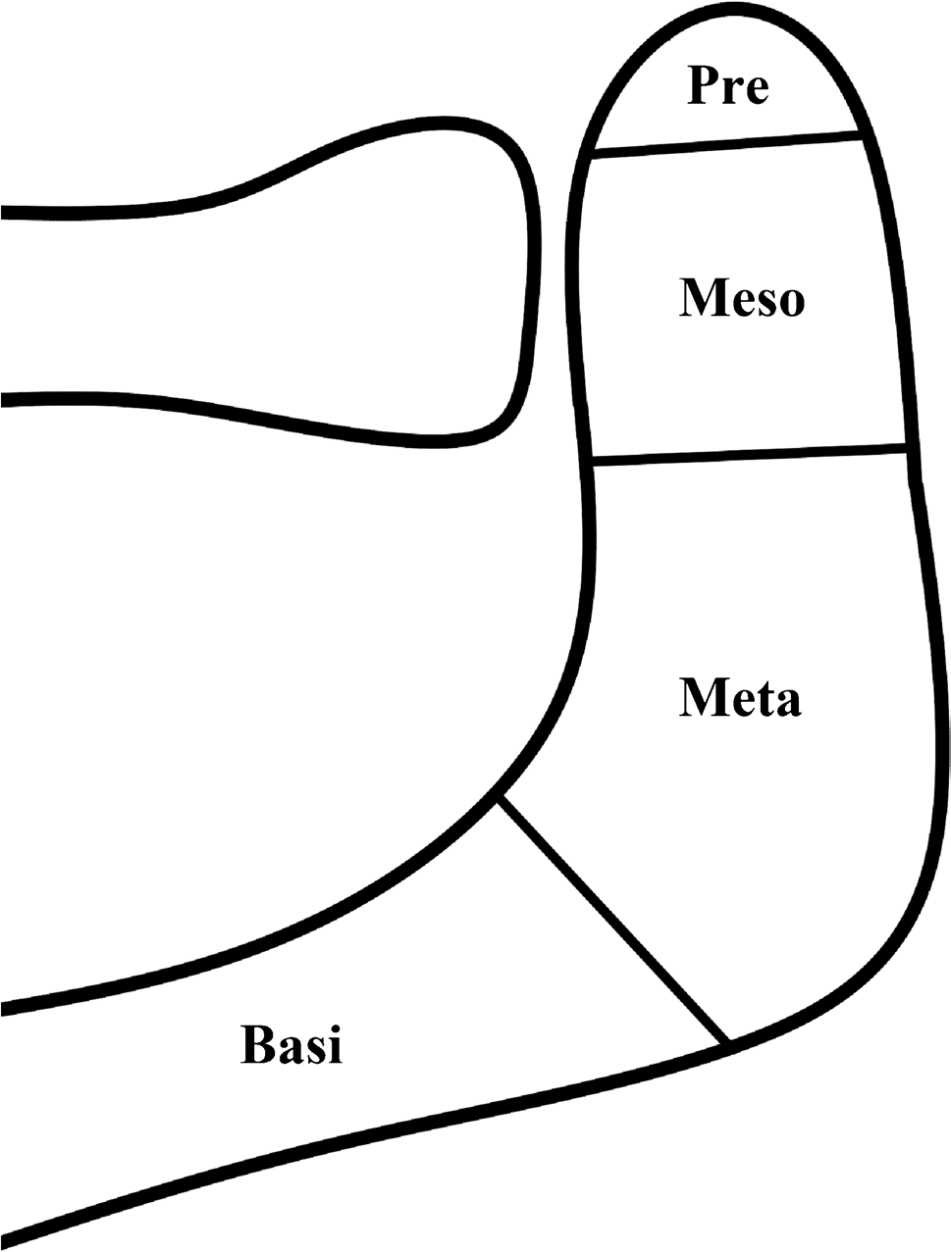
Scheme showing the types of os acromiale: pre-acromion, meso-acromion, meta-acromion, and basi-acromion

During fusion-failures, seven types of os acromiale are possible [7]. However, mostly three different os acromiale occur: the pre-acromion is the lack of fusion between pre- and meso-acromion, the meso-acromion between meso- and meta-acromion, and the meta-acromion between meta- and basi-acromion (Fig. 1) [8]. The most common form is the meso-acromion, followed by pre-acromions while meta-acromions are a rare condition [2, 8–13]. The attachment of the os acromiale to the rest of the acromion can be nearly complete and immobile, fibrocartilaginous (pseudarthrotic), or even a synovial joint [3, 9]. Os acromiale can be detected with radiographs, but the most reliable diagnostic is achieved by MRI [7].

The exact aetiology and associated factors of the os acromiale are not completely resolved. One hypothesis suggests a genetic predisposition [9, 14]. Another theory holds mechanical stress during the development of the acromion accountable [15]. A third option could be a combination of genetic and mechanic influences [4]. The reported prevalence of os acromiale ranges between 0.7 and 18.2% [4, 11] with a mean of 7% [12].

Regarding further associations, various results are described: association with the dominant side [4, 8, 11, 16, 17], gender [4, 8, 9, 11–13, 16, 17], or work load [4, 15] is discussed controversially. However, most of the present studies were conducted on cadavers, archeological skeletons, small or hospital-based samples. Therefore, population-based studies are crucial to clarify these topics.

In respect of the clinical relevance of the os acromiale, associations with various shoulder pathologies were described. Older studies determined an association between os acromiale and subacromial impingement as well as rotator cuff pathology and subacromial bursitis [7, 18, 19]. Recent studies are strongly questioning these associations [8, 11, 13, 20]. Even for patients with reverse shoulder arthroplasty (RSA), a comparable outcome to patients without os acromiale was described [21, 22].

However, sometimes os acromiale is a possible cause for pain [23]. There are different theories about the pathogenesis of symptomatic os acromiale: an inflammation at the pseudarthrosis, an unstable os acromiale leading to dynamic subacromial impingement, or arthritic degeneration due to hypermobility of the unfused bone [24, 25]. Regarding treatment of symptomatic os acromiale, there is no consensus until today.

Given the importance to not overlook an existing os acromiale, the lack of population-based values and knowledge on influencing factors, population-based studies are crucial. Therefore, the aim of this study was to assess the frequency, anatomy, and associations of os acromiale in a general adult population.

## Methods

### Design and sample

Data from the Study of Health in Pomerania (SHIP) was examined. The study is an ongoing population-based project with two independent cohorts, SHIP-START and SHIP-TREND. To achieve the population-based approach, participants were recruited randomly from official resident registry office files of a defined region in north-eastern Germany and stratified by sex, age, and city of residence. All participants were of Caucasian ethnicity.

As baseline (SHIP-START-0), 6265 eligible adults were chosen with a response of 68.8% in 1997. Between 2002 and 2006 (SHIP-START-1; *n* = 3300) and from 2008 to 2012 (SHIP-START-2; *n* = 2333), two follow-ups took place. The second cohort (SHIP-TREND-0, *n* = 4420) was examined baseline from 2008 to 2012. A high response rate was achieved by three written invitations, phone calls, and one personal contact. The local ethics commission approved the study and each participant gave written informed consent. Further study details have already been published [26].

**Fig. 2:**
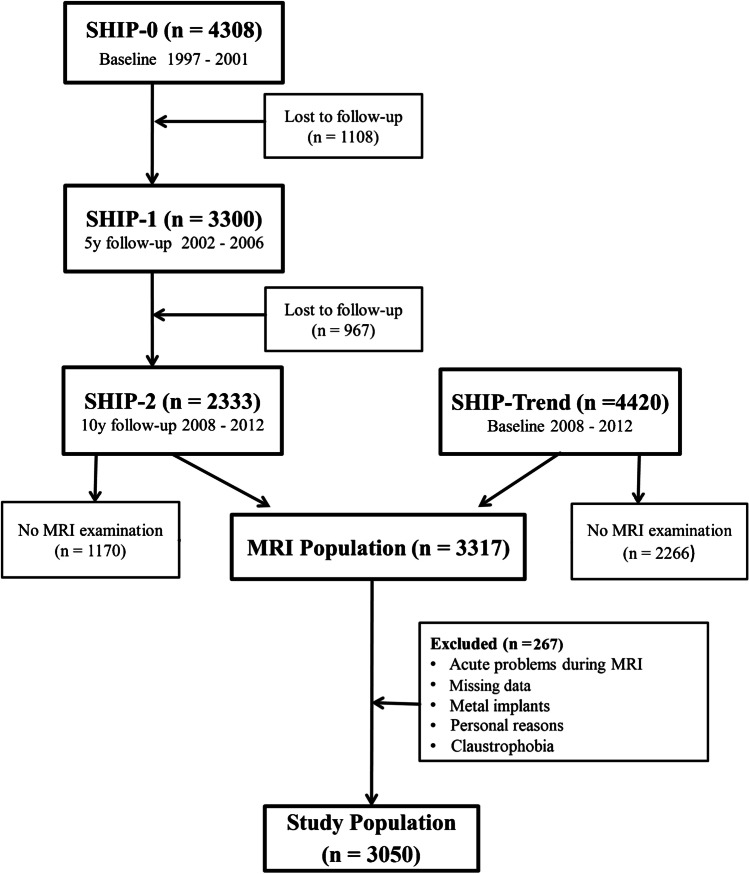
Flow diagram of the cohort from inclusion to the final study population

The presented analysis was performed on MRI of all participants from SHIP-START-2 and SHIP-TREND-0. 3317 of 6753 participants (SHIP-START-2 and SHIP-TREND-0) underwent the MRI examination, whereof 266 participants dropped-out because of claustrophobia, acute problems, metal implants, or personal reasons. One data was missing. In total, MRI of 3050 participants were investigated in the current analysis (Fig. 2).

### MRI protocol

The MRI was part of the standardized whole-body MRI (1.5-T MR scanner, Magnetom Avanto; Siemens Medical Systems, Erlangen, Germany). All MRIs were performed by four trained technicians. To assess the os acromiale, axial T1 volume interpolated breath hold examination sequences (repetition time 3.1 ms; echo time 1.1 ms; flip angle 8°; voxel size 1.8 × 1.8 × 3.0 mm), an axial T2 Half Fourier-Acquired Single shot Turbo spin Echo sequence (repetition time 550 ms; echo time 22 ms; flip angle 150°; voxel size 2.3 × 1.8 × 5.0 mm), and an coronal turbo inversion recovery magnitude sequence (repetition time 4891 ms; echo time 67 ms; flip angle 180°; voxel size 2.1 × 1.6 × 5.0 mm) were used.

### Image analysis

All MRI were examined by two trained examiners (CF, MF). The observers were blinded to all clinical information of the participants, while using the OsiriX (PIXMEO; Bernex, Switzerland) software. Since the axial plane is best to identify the “double joint” sign of an os acromiale [7, 8, 27], axial and coronal MRI in different thicknesses were used to perform all measurements and classifications. The presence and the type of an os acromiale were evaluated. Additionally, it was classified according to Gumina et al. [28] and its length, thickness, and width was measured in tenth of millimeter.

### Patient-related parameters

As patient-related parameters, the participants’ age, gender, body height, and body weight were determined. Additionally, shoulder pain on the visual analog scale (VAS) in the last seven days, proximal humeral fracture in the patients’ medical history, and the exposure to heavy mechanical oscillations on the upper extremity (e.g., working with impact drill machines, chainsaw) at the workplace and the amount of years were assessed. Moreover, seeing a surgeon or orthopedist in the last 12 months and the number of visits were documented.

### Statistics

Descriptive statistics such as mean values, standard deviations (SD), ranges, and percentiles were used to describe the sample. Student’s *t*-test was used for numerical variables; continuous variables were analyzed by Wilcoxon tests while categorical data was examined with Chi^2^-tests. A *p* value of < 0.05 was considered as statistically significant. Associations of demographic and anamnestic data with the os acromiale were analyzed by univariate logistic regression models. The statistical analysis was performed using Stata 16.1 (Stata Corp., College Station, TX, USA).

## Results

Out of the 3,050 participants 52% were females while the mean age was 52.8 ± 13.8 years with marginal differences between the sexes. The mean values of body height, body weight, and BMI were higher in males than in females (Table 1). Shoulder pain in the last 7 days was 1.1 (VAS) in the mean. Only 20 participants were left-handed.

**Table 1:** Sample characteristics

	*Total*	*Os acromiale*	*Male*	*Female*
*n*	3050	58	1460	1590
*Age [years]*	52.8 (13.8) [21–90]	53.9 (14.5) [24–78]	53.1 (14.4) [21–90]	52.5 (13.3) [21–88]
*Weight [kg]*	79.8 (15.1) [41.5–142.7]	86.3 (17.1) [45.6–125.3]	87.5 (12.9) [53.3–142.7]	72.7 (13.3) [41.5–126.1]
*Height [cm]*	169.9 (9.27) [137–202]	172.2 (9.72) [152–192]	176.5 (6.7) [156–202]	163.7 (6.7) [137–189]
*BMI [kg/m*^*2*^*]*	27.6 (4.43) [17.3–48.1]	29.0 (4.9) [18.3–39.0]	28.1 (3.7) [17.7–42.0]	27.2 (4.96) [17.3–48.1]

Data is presented as follows: mean (SD) [range]

In total, 72 os acromiale were found in 58 different participants. Therefore, 1.9% of the whole sample showed an os acromiale, while 21 (36%) were unilateral left, 23 (40%) were unilateral right, and 14 (24%) were bilateral. Sixty-eight meso-acromions, three pre-acromions, and one meta-acromion were detected (Table 2). The meso-acromions had a mean size of 23.16 mm (length) × 24.33 mm (width) × 10.31 mm (thickness). Detailed information on the size is presented in Table 3. Following the classification of Gumina et al. [28], 56.9% of all os acromiale were classified as intermediate, 36.1% were cobra shaped, 2.8% square tip, and in 4.2% the Gumina classification was not possible. In males, there were 12 os acromiale on the left side, 11 on the right side, and 13 men had a bilateral os acromiale. One of the male participants with an os acromiale had had a humeral head fracture in his medical history, but on the contralateral side. The frequency in women was 12 unilateral os acromiale on the right side while nine were unilateral left and one bilateral. Therefore, unilateral and bilateral os acromiale were more frequent for men than for women (right side: *p* = 0.037, left side: *p* = 0.005). No differences in sides were detected (*p* = 0.808). Two out of the 20 left-handed participants had an os acromiale but on the right side. Participants with os acromiale had a greater body height than participants without, but after adjusting the results on gender no significant differences were found (right side: *p* = 0.241, left side: *p* = 0.154). Gender-adjusted body weight analyses showed a significant association to right os acromiale (*p* = 0.016), whereas no significant results occurred for the left side (*p* = 0.132).

**Table 2:** Descriptive results of os acromiale

	*Total*	*Male*	*Female*
*n*	3050	1460	1590
*Unaffected*	2992	1424	1568
*Os acromiale right*	37 (1.21)	11 (0.75)^***#***^	12 (0.75)^***#***^
*Os acromiale left*	35 (1.15)	12 (0.82)^***#***^	9 (0.57)^***#***^
*Os acromiale bilateral*	14 (0.46)	13 (0.89)	1 (0.06)
*Pre-acromion*	3 (4.2)	3 (5.8)	0 (0)
*Meso-acromion*	68 (94.4)	48 (92.3)	20 (100)
*Meta-acromion*	1 (1.4)	1 (1.9)	0 (0)

Data is presented as follows: amount (frequency in %)

^#^Unilateral

**Table 3:** Mean size of the os acromiale

	*Length (mm)*	*Width (mm)*	*Thickness (mm)*
*Pre-acromion (n* = *3)*	9.0 (1.5) [7.5–10.5]	13.9 (3.3) [11.8–17.7]	7.0 (10.8) [6.3–7.9]
*Meso-acromion (n* = *68)*	23.2 (3.7) [14.9–29.6]	24.3 (2.8) [16.9–29.2]	10.3 (1.8) [7.3–13.6]
*Meta-acromion (n* = *1)*	38.7	22.5	9.6

Data is presented as follows: mean (SD) [range]

No associations to participants’ age (right: *p* = 0.993, left: *p* = 0.499), shoulder pain (right: *p* = 0.559, left: *p* = 0.445), visiting a surgeon (right: *p* = 0.787, left: *p* = 0.367) or orthopedist (right: *p* = 0.600, left: *p* = 0.481), and to heavy mechanical oscillations on the upper extremity (right: *p* = 0.054, left: *p* = 0.117) were detected.

## Discussion

The os acromiale is mostly investigated by studies on skeletons or cadavers. Only few authors published results in vivo using hospital-based data to conduct these studies (Table 4). In addition, the lack of population-based knowledge on frequency and influencing factors is still ongoing. For this reason, we decided to investigate MRI of the population-based SHIP project.

In this large population-based study, 1.9% of the 3050 participants had at least one os acromiale. The frequency of bilaterality was 24%.

**Table 4:** Frequency of os acromiale, compared to other studies’ normal/control group

Author year	Method	Population	Age	*N*	Os acromiale (%)	Rate of bilateral (%)
Macalister 1893 [3]	Scapular bones	UK	–	100	15 (15)	–
Vallois 1926 [17]	Scapular bones	French	–	292	8 (2.7)	1 (14.3)
Liberson 1937 [30]	Radiographs	USA	–	1800	(2.7)	(62)
Nicholson 1996 [10]	Scapular bones	USA	[21–70]	210	17 (8)	7 (41)
Sammarco 2000 [31]	Scapular bones	USA	44.7 [18–89]	1033 M	(8.5)	(35.2)
				165 F	(4.9)	(12.5)
Gumina 2003 [28]	Radiographs	Italy (asymptomatic control group)	54 [29–79]	222	11 (4.9)	–
Boehm 2005 [13]	Radiographs (rotator cuff repairs)	Germany	55	1000	62 (6.2)	–
Case 2006 [4]	Cadaver skeletons	South African	> 18	494	(18.2)	36
	Medieval skeletons	Danish		532	(7.7)	8
Coskun 2006 [29]	Radiographs	Turkish	–	90	1 (0.9)	–
	Scapular bones			90	1 (0.9)	
Hunt 2007 [9]	Skeletons	USA black	> 25	481 M	60 (12.47)	29 (48.3)
				347 F	32 (9.22)	15 (46.9)
		USA white		456 M	31 (6.80)	9 (29)
				310 F	10 (3.23)	4 (40)
Kumar 2013 [11]	Radiographs/MRI	Korean	55.7 [21–91]	1568	13 (0.7)	2 (15.4)
Rovesta 2017 [8]	MRI	Italy	55.8 [25–91]	726	25 (3.44)	–
Aibinder 2017 [16]	Primary RSA	USA	72.0 [46–84]	1079	25 (2.3)	–
Present study	MRI	Germany	53	1460 M	36 (2.5)	13 (36.1)
			52	1590 F	22 (1.4)	1 (4.6)
			53	3050 T	58 (1.9)	14 (24.1)

*M* male, *F* female, *T* total. Age is presented in years as follows: mean [range]

As described in earlier studies [8–12], most os acromiale in the present study were os meso-acromiale (94.4%). Compared to previous studies on os acromiale (Table 4), frequency and proportion of bilaterality in the current study were considerably lower. In their review, Yammine described a prevalence of 5.78% in 3935 individuals, respectively 5.16% at the pooled rate from seven large-sample studies for os acromiale for people of white ancestry [12]. However, the prevalence varies depending on the ethnic group. Asian studies reported the lowest frequency with 0.7% [11]. The prevalence in Caucasians varies between 1 and 15% [3, 29, 30] while studies in African-Americans and Africans had the highest prevalence with 9.2–18.2% [4, 9]. Bilateral os acromiale were reported with a frequency of 12.5–62% [30, 31]. Additionally, a higher frequency of bilateral os acromiale is described for black people [9, 31].

Since Angel et al. [14] proposed the idea of a genetic influence on the formation of os acromiale due to their results on the remains of a Baptist graveyard in 1987, multiple studies supported this view [9, 12, 31]. An opposing theory was suggested by Stirland which considers mechanical stress as the main cause for the formation of os acromiale due to their observations on the British warship Mary Rose. On the basis of the high number of longbow archers and the high prevalence of os acromiale, Stirland proposed the theory of stress-induced trauma [15]. In opposition, Case et al. did not find any differences between people with higher and lower socioeconomic status, while for a lower socioeconomic status harder physical work was presumed [4]. The results of the present population-based study mainly support the genetic theory because no association between os acromiale and high exposure to heavy mechanical oscillations on the upper extremity was found. Additionally, with 1.9% a lower prevalence of the os acromiale was detected even though participants starting from the age of 18 were included. Consequently, falsely diagnosed os acromiale could possibly be included in our sample because they would have moved on to consolidation later in life. However, our exclusively Caucasian sample of north-eastern Germany seems to have a distinct genetic pool with a low rate of os acromiale compared to the worldwide prevalence of 7% [12] and therefore the sample’s low prevalence supports the genetic theory.

Moreover, no differences regarding sides were detected. A conclusive calculation regarding the dominant side was not possible because only 20 left-handed participants were present in our study sample. However, a correlation to the dominant side seems unlikely because the two left-handed participants with os acromiale were os acromiale on the right side. In previous works, Aibinder et al. discovered os acromiale more frequently on the dominant side [16], while other authors found higher frequencies on the left side [4] or on the right side [8]. Some studies did not find any differences in sides [11, 17].

Gumina et al. suggested in 2003 that the frequency of os acromiale increases with increasing distance between the anterior aspect of the acromion and the acromioclavicular joint [28]. However, the present results cannot support this thesis as most of the os acromiale were intermediate shaped according to the Gumina classification (56.9%).

Regarding associations to os acromiale, a higher prevalence for men was detected, while no associations for age and body height were present. The significant association between body weight and the os acromiale on the right side seems to be coincidentally. Previous studies revealed a higher prevalence of os acromiale in men [9, 16] as well but many authors did not confirm the gender differences [4, 8, 11–13, 17]. Associations to age were not found until now [4].

Given that stable and asymptomatic os acromiale can become unstable and symptomatic due to trauma or surgical interventions, e.g., subacromial decompression [32, 33], it is crucial to be aware of potential os acromiale before surgery. In addition, the os acromiale can be a cause of shoulder pain itself due to inflammation at the pseudarthrosis, dynamic subacromial impingement, or arthrosis [24, 25]. Therefore, it is of clinical relevance to recognize os acromiale and be aware of its prevalence.

Regarding treatment of symptomatic os acromiale, there is no consensus until today. Usually, a conservative approach is used as first-line therapy for at least six months. Afterwards, surgical intervention should be considered [34]. The discussion about the optimal surgical technique is still ongoing. Currently different methods like excision, acromioplasty, and open reduction and internal fixation (ORIF) are the topic of many reviews. With small os acromiale, there is a trend to excision, while for larger os acromiale ORIF seems to be beneficial to preserve the deltoid function [25, 32, 34].

A limitation of our study might be how representative our sample is. Due to selection by response to the baseline SHIP-TREND-0, to the baseline and follow-up examinations of SHIP-START-2 as well as to the MRI subproject, representativeness might have been diminished. Moreover, our study consists of an exclusively Caucasian population. With respect to known ethnic differences [9, 12], our results may not apply to non-Caucasian individuals. Another limitation is the possible inclusion of participants with shoulder pathology through the population-based study design. However, none of our participants with os acromiale showed any sign of previous shoulder surgery (metal implants, anchors etc.). One participant with os acromiale had a humeral fracture in his history, but on the contralateral side. Moreover, we investigated a general population, so it can be expected that the majority of the participants were healthy and without pathology. Since participants with metal implants are excluded due to the MRI, the prevalence could be underestimated. Another weakness of the current study is the cross-sectional design, which only provides associations, but no cause-and-effect relationships.

A last limitation might be the already mentioned inclusion of participants from the age of 18 which could lead to a degree of bias with falsely diagnosed os acromiale since some authors stated that the os acromiale should only be diagnosed after the age of 25 [18]. However, recent studies showed that the complete osseous fusion of the acromion takes place before the age of 18 [5, 6]. In MRI on 85 children, Kothary and Rosenberg detected a starting fusion of the ossification centers at the age of 14. Generally, it was completed after the age of 16 [5].

## Conclusion

In conclusion, the prevalence of 1.9% of os acromiale in north-eastern Germany is relatively low comparing to the worldwide prevalence of 7%. This may be based on the genetic pool in this area. Additionally, no other association to lifestyle, age, gender, or sides was detected so our study supports the genetic theory for the etiology of the os acromiale. Despite the low prevalence, it is important to be aware of potential os acromiale before surgery, so MRI are advised. Since therapy on symptomatic os acromiale depends on the size of the os acromiale, current measurements were given.
